# Thermal Insulation and Sound Absorption Properties of Open-Cell Polyurethane Foams Modified with Bio-Polyol Based on Used Cooking Oil

**DOI:** 10.3390/ma13245673

**Published:** 2020-12-12

**Authors:** Maria Kurańska, Roman Barczewski, Mateusz Barczewski, Aleksander Prociak, Krzysztof Polaczek

**Affiliations:** 1 Department of Chemistry and Technology of Polymers, Cracow University of Technology, Warszawska 24, 31-155 Cracow, Poland; aleksander.prociak@pk.edu.pl (A.P.); krzysztof.polaczek@doktorant.pk.edu.pl (K.P.); 2 Faculty of Mechanical Engineering, Institute of Applied Mechanics, Poznan University of Technology, Jana Pawła II 24, 60-965 Poznan, Poland; roman.barczewski@put.poznan.pl; 3 Faculty of Mechanical Engineering, Institute of Materials Technology, Poznan University of Technology, Piotrowo 3, 61-138, Poznan, Poland; mateusz.barczewski@put.poznan.pl

**Keywords:** open-cell polyurethane foams, bio-polyol, sound absorption properties, circular economy, thermal insulation

## Abstract

The main goal of this work was to evaluate the thermal insulation and sound absorption properties of open-cell rigid polyurethane foams synthesized with different contents of cooking oil-based polyol. The content of the applied bio-polyol as well as flame retardant (triethyl phosphate) in the foam formulation had a significant influence on the cellular structures of the materials. The open-cell polyurethane foams were characterized by apparent densities in the range 16–30 kg/m^3^. The sound absorption coefficients of the polyurethanes with various contents of bio-polyol were determined using the standing wave method (Kundt’s tube) in the frequency range of 100–6300 Hz. The effect of the content of the bio-polyol and flame retardant on the coefficient of thermal conductivity (at average temperatures of 0, 10 and 20 °C) as well as the compressive strength (at 20 and −10 °C) was analyzed. Different trends were observed in terms of the thermal insulation properties and sound absorption ability of the open-cell polyurethanes due to the addition of bio-polyol. In conclusion, it is necessary to use systems containing both petrochemical and bio-based raw materials.

## 1. Introduction

Polyurethanes (PUR) and other polymers are based on petroleum feedstock. However, vegetable oils as well as industrial waste have been used more and more to replace petroleum following the principle of circular economy: to retain resources and products in use [1,2,3,4].

Industrial waste reduction is a fundamental task within sustainable development. Recycling and valorization of used cooking oil (UCO) can play a major role in environmental protection. UCO is traditionally used for bio-fuel production, yet, the popularity of bio-diesel in the automotive industry seems to be decreasing. The current global production of UCO is estimated as between 20% and 32% of the total vegetable oil consumption (41–67 Mt/year). The main sources of UCO are households and hospitality sectors (hotels, restaurants, casinos, cafes, catering) [5].

The valorization of UCOs via chemical modification into suitable oleochemical products such as plasticizers, binders and lubricants has drawn the attention of academic and industrial researchers over the last two decades [5,6,7]. Our recent results show that UCOs can be also applied instead of refined oil in the synthesis of epoxidized oils and polyols thanks to the presence of unsaturated bonds in their chemical structures. The epoxidized UCOs that we obtained were characterized by epoxy values in the range 0.10–0.34 mol/100g [8]. Bio-polyols synthesized by ring opening reactions were characterized by hydroxyl values of 100–295 mgKOH/g [9,10,11]. Such a wide range of hydroxyl values allows applications of bio-polyols in the preparation of different types of polyurethane foams (rigid, semi-rigid, flexible). Other methods used in the synthesis of polyols from waste materials include oxypropylation and acid liquefaction of bio-mass residues as well as polymerization of crude glycerol, which is a byproduct of bio-diesel production [1,12,13].

Bio-polyols based on waste resources can successfully replace petrochemical polyols in the production of PUR. However, given their various chemical structures, it is necessary to analyze the influence of bio-components on the foaming process and properties of PUR materials. Most of the studies described in the literature concern the influence of bio-components on the physical and mechanical properties and thermal stability of PUR foams with apparent densities higher than 30 kg/m^3^. In the literature, there are a few articles describing the modification of open-cell PUR foams with an apparent density lower than 20 kg/m^3^ modified with bio-based polyols. One of such works concerns the influence of the hydroxyl values of bio-polyols on the reactivity, cellular structures, mechanical and thermal properties of PUR systems [11]. It concludes that the high viscosity of the bio-polyols caused by a higher content of oligomers makes the foaming process difficult, which is a key factor especially in the case of open-cell foams with very low apparent densities. The most beneficial properties were obtained for the foams modified with the bio-polyol characterized by hydroxyl value 200 mgKOH/g and viscosity ca. 4000 mPa∙s [11]. It is also possible to apply bio-polyols with a lower hydroxyl value. In another work [14] bio-foams based on bio-polyols characterized by hydroxyl values 140 and 159 mgKOH/g are described. Significant differences were found in the case of bio-polyol viscosities of 3275 and 961 mPa∙s. However, it was concluded that the differences in viscosity and hydroxyl values in such a range did not affect the properties of the foams with a low apparent density significantly.

In the literature, there have been few studies discussing the influence of bio-components on the acoustic properties of open-cell PUR foams. PUR foams are generally used in thermal insulation materials but their porous structure allows for their use as sound absorbers. It is possible due to cavities, channels or interstices present in their structure. The frictional forces between the air flow and cell walls convert the sound energy into heat when sound waves flow through an open pore [1]. Gama et al. have described the sound absorption properties of rigid polyurethane foams produced from crude glycerol (CG) and/or liquefied coffee grounds-derived polyol (POL). Due to a different lignin content, the POL-based foams had slightly higher sound absorption coefficient values at lower frequencies while the CG foams had higher sound absorption coefficient values at higher frequencies [1]. The presence of lignocellulosic materials increased cell sizes of the foams and their stiffness, which increased the sound absorption at low frequencies. The lower stiffness of the foams based on CG improved their acoustic properties at higher frequencies. However, the PUR formulation has an important influence of the foaming process and cell structure and as a consequence on the sound absorption ability (at various frequencies) of foams. The behavior of PUR foams modified by varying the weight proportions of the main components (polyol and isocyanate) and foams with fixed ratios of polyol to isocyanate but different apparent densities was analyzed by [15]. They revealed that an increased polyol content gives rise to improved sound absorption behavior, particularly in the medium and high frequency ranges. It was also found that the improved mechanical energy dissipation behavior went alongside the reduced acoustic energy absorption performance, which might be attributed to the cell structure of the investigated foams. This work focuses on the relationship between the content of bio-polyol based on waste cooking oil and the mechanical and acoustic properties of bio-foams. Additionally, the effect of flame retardant, which also acts as plasticizer of the PUR matrix, on the mechanical and acoustic properties is discussed.

## 2. Materials and Methods

### 2.1. Materials

Bio-polyol with a hydroxyl number of 270 mgKOH/g was synthesized from used cooking oil at Cracow University of Technology according to the method described in our earlier publications [9,16]. A petrochemical polyether polyol with the trade name Rokopol RF-551 and a hydroxyl number of 440 mgKOH/g was supplied by PCC Rokita (Brzeg Dolny, Poland). Polymeric methylene diphenyldiisocyanate (Ongronat 2100) containing 31.5 wt.% of free isocyanate groups was supplied by Borschodchem (Kazincbarcika, Hungary). Catalysts of foaming (Polycat 37) and gelling (Kosmos 19) reactions as well as surfactants (Tegostab 8870 and Ortegol 500) were provided by Evonik (Essen, Germany). Carbon dioxide (chemical blowing agent) was generated in the reaction of water with isocyanate groups. The flame retardant (FR) triethyl phosphate (TEP) was provided by LANXESS Deutschland. The content of each component and the isocyanate indexes are shown in Table 1.

### 2.2. Preparation of Samples

Open-cell polyurethane foams with different contents of the bio-polyol (20–100 wt.%) were prepared using a one-step method from components A and B. Component A consisted of polyols, catalyst, surfactant, water and flame retardant. This composition was stirred for 30 s and then isocyanate (component B) was added. Next, the system was mechanically mixed for 5 s and poured into an open mold. The foams were kept at room temperature for 24 h of seasoning.

### 2.3. Characterization of Foam Properties

The morphology of cells was analyzed using a scanning electron microscope (SEM) TM3000 (Hitachi, Tokyo, Japan) and the software ImageJ (version 1.53f). National Institutes of Health, Bethesda, MD, USA) was used for image analysis. Five images were analyzed. The closed cell content in the foams was measured according to the ISO 4590 standard [17]. The contents of closed cells in those five samples were measured and averaged. The apparent density of the PUR foams was determined as the ratio of the sample weight to its volume following the ISO 845 standard [18]. The samples were measured and weighed to an accuracy of 0.01 mm and 0.01 g, respectively.

The thermal conductivity coefficients were determined using a Laser Comp Heat Flow Instrument Fox 200 (TA Instruments, New Castle, DE, USA) and foam samples with dimensions of 5 cm × 20 cm × 20 cm [19]. The thermal conductivity was analyzed at average temperatures of 0, 10 and 20 °C (the temperature of the cold plate was −10, 0 and 10 °C, respectively, and the temperature of the warm plate was 10, 20 and 30 °C, respectively). The thermal conductivity coefficients of three samples were measured and averaged.

The compressive strength (σ) was measured at 10% deformation using a Zwick 1445 instrument (Zwick Roell Group, Ulm, Germany) for samples with dimensions of 5 cm × 5 cm × 5 cm according to PN-EN 826 [20]. The compressive strength of five samples were measured and averaged. The compressive force was applied at a speed of 2 mm/s, axially in a perpendicular direction to a square surface. The compressive stress was calculated at 10% deformation.

**Figure 1:**
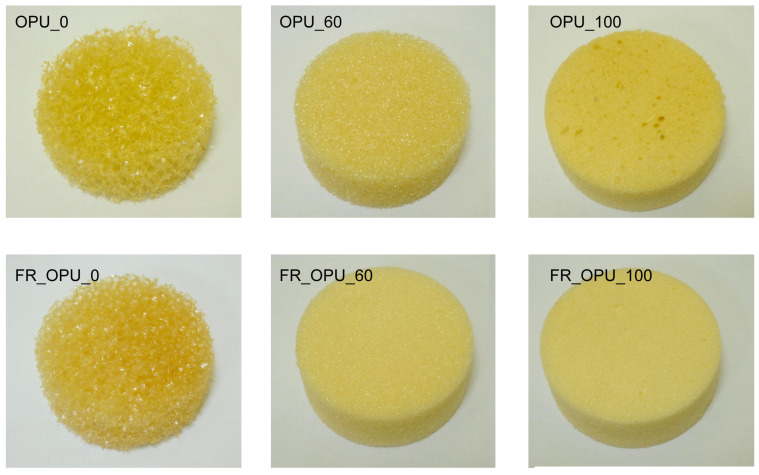
Photographs of 100 mm-diameter polyurethane (PUR) samples used for acoustic properties evaluation in the frequency range of 100–1000 Hz.

The sound absorption coefficients of the new porous materials were determined using the standing wave method EN ISO 10534-1 standard [21,22]. This method does not require testing in special chambers. The use of a standing wave apparatus enables the determination of the sound absorption coefficient under well-defined and controlled measurement conditions [23]. The sound absorption coefficients were determined in 1/3 octave bands (center frequencies of 1/3 octave bands): 100, 125, 160, 200, 250, 315, 400, 500, 630, 800 Hz and 1, 1.25, 1.6, 2, 2.5, 3.15, 4, 5, 6.3 kHz. Two measuring tubes were applied in the test. A tube with a diameter of 100 mm was used for testing in the 1/3 octave bands from 100 Hz to 1 kHz, while a tube with a diameter of 30 mm was used in the 1/3 octave bands from 1.25 kHz to 6.3 kHz. Taking into account the abovementioned measurement methodology, the samples for acoustic tests were cut into cylindrical shapes with diameters of 100 mm and 30 mm and heights of 40 mm. Figure 1 presents collectively the photographs of 100 mm-diameter samples used for acoustic properties evaluation.

**Figure 2:**
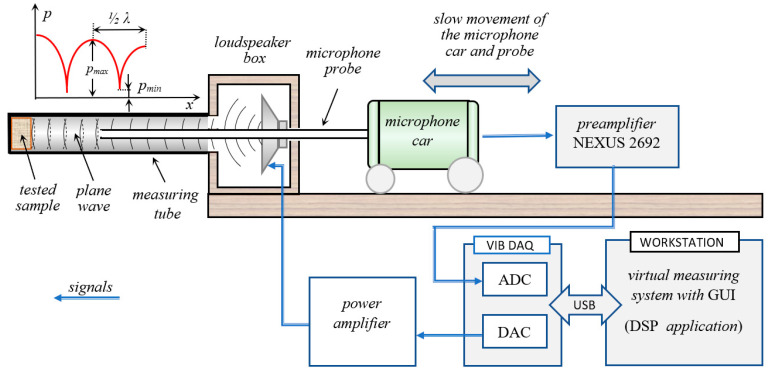
Diagram depicting measurement system used to determine sound absorption coefficients.

The measuring system used to conduct the tests was partially based on elements of a 4002 (B&K) Standing Wave Apparatus (such as measuring tubes, loudspeaker and microphone probe) [24]. However, a low-noise preamplifier NEXUS type 2692 (Brüel&Kjær, Nærum, Denmark), a power amplifier type A5512 (Radmor, Gdynia, Poland), a 24-bit VibDaq 2+ signal acquisition module (EC Electronics, Kraków, Poland) and a digital signal processing application were used to create a hybrid signal conditioning and signal processing system. The digital signal processing application was developed in the DASYLab^®^ programming environment (Data Acquisition System Laboratory Ver. 7, DASYTec, Amherst, NH, USA, a National Instruments Company). A simplified diagram of the measurement system used to determine the sound absorption coefficients is shown in Figure 2.

It is worth mentioning that the digital signal processing procedures included:

- generation of monoharmonic signals transmitted through the power amplifier to the loudspeaker,
- narrowband filtering of the signal recorded by the microphone probe to remove interference and noise (Butterworth filters; filter order *n* = 10); the center frequency of the filter was consistent with the frequency of the generated monoharmonic signal,
- precise tracking of changes in pressure when moving the microphone probe and automatic detection and storage of maximum and minimum pressure values; precise measurement of the minimum local pressure was crucial especially in the frequency bands where the absorption coefficient had low values (below 0.1),
- graphical user interface (GUI).

Monoharmonic (sinusoidal) signals were generated digitally. Frequencies of the test signals were equal to the center frequencies of the 1/3 octave bands (in the range from 100 Hz to 6.3 kHz). A loudspeaker powered by an amplifier produced an acoustic plane wave that propagated down the tube and reflected from the sample. The absorption coefficient can be calculated on the basis of the measurements of the minimum and maximum acoustic pressure (*p_min_* and *p_max_*) of a standing wave, which is set up within the tube (see Figure 2).

The acoustic absorption coefficient was determined using the formulas [23,24]:

(1) $$\alpha =1-{\left(\frac{n-1}{n+1}\right)}^{2}$$

where *n* is the standing wave ratio defined as:

(2) $$n=\frac{p_{max}}{p_{min}}$$

and *p_max_, p_min_* are the maximum and minimum pressures in the tube.

An additional parameter, which offers a useful evaluation of the sound absorption ability for engineering purposes, is the average sound absorption coefficient (*α_avg_*) [25]. Based on the values of the absorption coefficients in the 1/3 octave bands, the *α_avg_* value can be calculated from the following formula:

(3) $$\alpha_{avg}=\frac{1}{n}\overset{n}{\underset{i=1}{\sum }}\alpha_{f\left(i\right)}$$

where *α_f_*(*i*) are the sound absorption coefficients measured at the center frequencies *f*(*i*) (e.g., 100 Hz… 6.3 kHz) and n is the number of 1/3 octave bands taken into account.

## 3. Results

### 3.1. Cellular Structure and Thermal Conductivity

**Figure 3:**
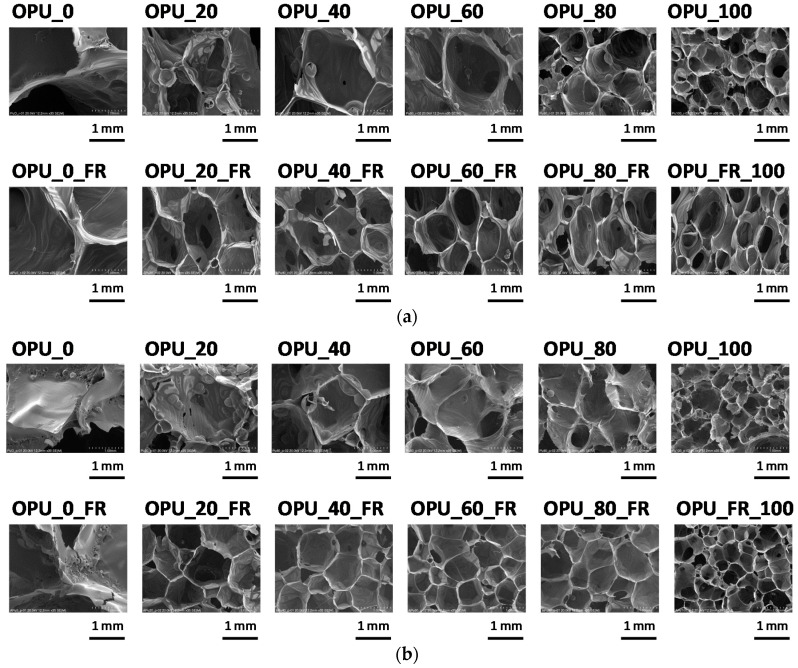
Cellular structure of foams modified with bio-polyol: (**a**) cross-section of the area parallel to foaming direction (**b**) cross-section of the area perpendicular to foaming direction.

The size and shape as well as the content of open cells are important parameters of foam cellular structure, which is directly related with thermal insulation and mechanical properties of a PUR foam. The influence of the used cooking oil-based polyol on the cellular structure of the PUR foams was examined using SEM (Figure 3). The average equivalent diameter and content of closed cells are listed in Table 1.

In the case of most samples, it was observed that the equivalent diameter decreased when the bio-polyol content increased. Additionally, this tendency was strengthened through the modification of the PUR formulation with the flame retardant. This effect was most visible in the case of cells where the cross-section area was measured perpendicularly to the foam rise direction. In our previous work, we concluded that replacing the petrochemical polyol with 60% of the bio-polyol in closed-cell PUR foams resulted in the formation of an increased number of small-area cells [10]. Based on the current research, it was confirmed that bio-polyols generally led to a decrease of the cell size regardless of the type of foams (closed or open-cell). This effect can be caused by the chemical structure of the bio-polyol, where hydroxyl groups are located in the middle of the alkyl chains of triglycerides. Hydroxyl derivatives of natural oil act as a surfactant with the ability to lower the surface tension due to the presence of ester groups, ether diethylene glycol chains and hydroxyl groups as hydrophilic parts as well as hydrocarbon chains of fatty acids as hydrophobic parts. Additionally, the flame retardant can also act as a surfactant and cause a decrease of the surface tension, which is confirmed by the lowest cell diameters of the foams where the petrochemical polyol was fully replaced with the bio-polyol.

The cellular structure as well as the apparent density of the foams significantly affect their thermal conductivity. Thermal conductivity (λ) is an important property that determines the insulation properties of PUR foams [26]. The coefficient of thermal conductivity of the open-cell PUR foams was measured at three different average temperatures (0, 10 and 20 °C). The results are listed in Table 2.

The coefficient of thermal conductivity of commercial open-cell polyurethane foams (at an average measurement temperature of 10 °C) is in the range of 0.037–0.039 W/(m·K) and is much higher than in the case of closed-cell foams (0.020–0.022 W/(m·K)) [11]. In this work, the thermal conductivity of the materials modified with the bio-polyol and TEP was comparable to commercial PUR foams regardless on the bio-polyol content. In the case of the foams without the flame retardant, beneficial values of thermal conductivity were obtained for the materials modified with 80% and 100% php of the bio-polyol. However, these materials had higher apparent densities, which is not typical of spraying PUR foams. This is an effect of the cellular structure of the materials, which depends strongly on the viscosity of the reaction mixture [2]. The increasing density of the polyol premix as a result of introducing higher and higher contents of the bio-polyol with higher viscosity compared to the petrochemical polyol resulted in further inhibition of cell growth [27]. This is why the apparent density of OPU_80 and OPU_100 is higher compared to the foams with lower contents of the bio-polyol. In the case of the flame retardant-modified foams, the materials had similar densities. The addition of the flame retardant reduced the viscosity of the polyol premix and did not limit the foam growth. Based on these results, it was concluded that the increase of the apparent density had a beneficial effect on the coefficient of foams thermal conductivity. It was shown that a finer cellular structure had a decisive influence on the thermal conductivity of the final open-cell foams regardless of the measurement temperature.

**Figure 4:**
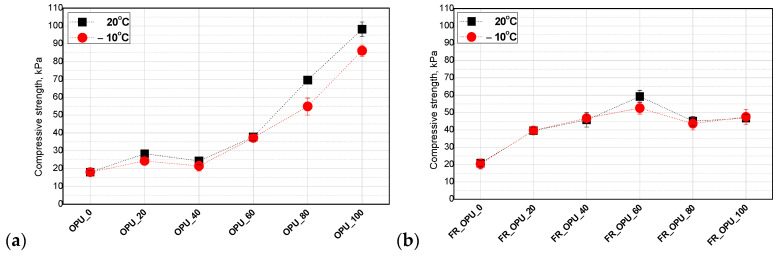
Influence of bio-polyol (**a**) and both bio-polyol and flame retardant (**b**) on compressive strength of open-cell PUR foams.

The alkyl dangling chains of the fatty acids in bio-polyol also have an influence on the compressive strength of porous materials. They can act as plasticizers reducing the polymer rigidity while increasing its flexibility [28]. Such an effect is especially visible in the case of PUR foams with higher density (>35 kg/m^3^). For open-cell foams with apparent densities below 20 kg/m^3^, the modification of the PUR formulation with bio-polyols leads to an increase of compressive strength, especially when the petrochemical polyol was replaced with 40–60 wt.% of the bio-polyols [29]. Figure 4 shows the values of the apparent density and compressive strength of the open-cell PUR foams prepared in the experiment modified with different amounts of the bio-polyol based on used cooking oil.

Compressive strength ranged between 20–100 kPa and 20–60 kPa for the foams without and with TEP, respectively. It was concluded that the measurement temperature does not have a significant influence on the mechanical properties of the foams and such materials can also be used at temperatures below 0 °C. A correlation between the bio-polyol content and both the compressive strength and apparent foam density was observed. The higher the apparent density, the higher the compressive strength of OPU_80 and OPU_100. Gosz et al. observed a similar effect in the case of polyurethane–polyisocyanurate foams. In that work, the compressive strength and apparent density ranged between 105 and 375 kPa and 39–43 kg/m^3^, respectively [30].

**Figure 5:**
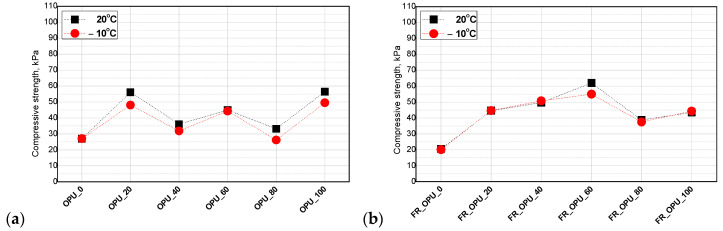
Normalized compression strength of PUR foams modified with bio-polyol (**a**) and bio-polyol and flame retardant (**b**).

In order to eliminate the influence of the higher apparent density on the mechanical properties of the foams, the normalized compressive strength was determined according to the equation of Hawkins [31]. The mechanical properties were normalized to the average PUR foam apparent density (*ρ_norm_*) for the foams with TEP and without TEP, 19 and 22 kg/m^3^ respectively. The equation for the normalized compressive strength calculation is presented below:

(4) $$\sigma_{norm}=\sigma_{exp}{\left(\frac{\rho_{norm}}{\rho_{sample}}\right)}^{2.1}$$

where *σ_exp_* is the experimentally determined compressive strength and *ρ_sample_* is the PUR sample apparent density (Figure 5).

A calculation of the normalized values of compressive strength allows us to evaluate the influence of PUR system modifications independently of the foam’s apparent density. In the cases considered in this work, it was noticed that the use of the bio-polyols in a broad range to replace the petrochemical polyol did not worsen the compressive strength of the modified foams. Moreover, the increase of the compressive strength shown in Figure 4a is generally the effect of the apparent density increase. On the other side, the foams containing the flame retardant exhibited a similar character of changes to those of non-normalized values. It means that the plasticization effect of the flame retardant is reduced by the application of the bio-polyol as a replacement of the petrochemical polyol. The mechanical strength of these materials increased substantially after the replacement of the petrochemical polyol by up to 60%. The value of the compressive strength was slightly reduced, however, it was still much higher compared to the reference material.

### 3.2. Acoustic Properties of PUR Foams

**Figure 6:**
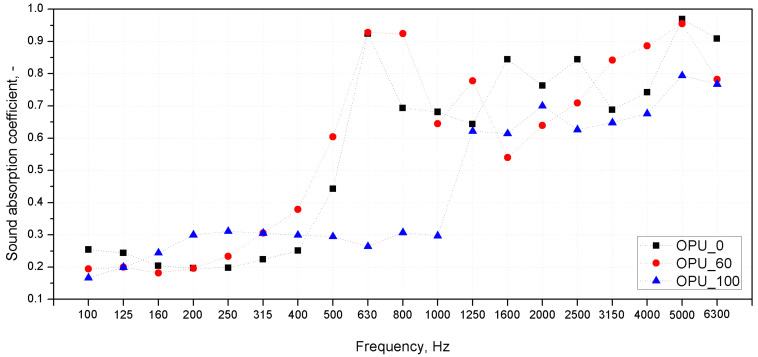
Sound absorption coefficients of OPU PUR foams.

**Figure 7:**
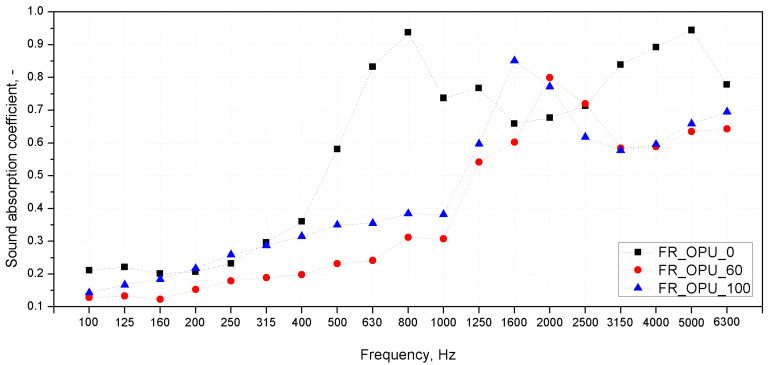
Sound absorption coefficients of FR_OPU PUR foams.

According to the literature, the sound absorption coefficient of cellular materials, including polyurethane foams, is closely related to the size of the cells and their structure [32]. The ability of a foam to suppress sound in selected frequency ranges depends on the number of open cells in the foam and the size of the cells [33]. The sound waves induce vibrations in thin-walled cellular structures and the air inside them. Damping of the vibrations by the cell walls subjected to micro-deformations affects the presence of the frictional forces between the airflow and the cell wall which converts the sound energy into heat. As a result of greater deformation of cell walls observed for foams with larger cell sizes, a significantly greater amount of sound waves are converted into kinetic energy, leading to a more effective sound attenuation phenomenon [34]. In our experiment, because of a comparable content of open cells in all the PUR series, the crucial effect on the final acoustic properties of the foams is related to the cell size. For the acoustic attenuation evaluation, three series of materials were selected from among the unmodified and FR-containing foams. The acoustic properties were assessed for the foams without the addition of bio-polyol (OPU_0), the foams with a dominant bio-derivative material (OPU_60) and those produced only with its participation (OPU_100). A comparison of the sound absorption coefficients in 1/3 octave bands (presented as a function of center frequencies of these bands) for selected polyurethane foams with and without a flame retardant are presented in Figure 6 and Figure 7. The α(f) graphs present the results of the acoustic properties evaluation using Kundt’s tube method in the range of 100–6300 Hz, for samples with 100 and 30 mm diameters. The samples were placed in an experimental stand and tested parallel to the direction of foam growth. It means that the direction of acoustic waves propagation within the tube was consistent with the direction of foam growth. All the materials under investigation showed sound absorption coefficient changes typical of polyurethane foams, an increase of α in the range between 400 and 1000 Hz resulting from micro-resonances of thin-walled flexible structures [33]. It can be seen that the cell size measured and presented in Table 3 has a direct impact on the sound absorption capacity of the foams in the lower frequency range (below 500 Hz). The foams without FR containing 0 and 60% of bio-polyol showed a significant increase in α above 500 Hz. A similar effect was noted for a foam from the FR_OPU series without the bio-based polyol additive. These results are consistent with the literature data and the cell size change results [32,33]. Interestingly, in the case of FR-OPU_0, the foams were characterized by smaller cell sizes than OPU_0, but at lower sound frequencies (from 160 to 500 Hz) they had slightly higher values of the sound absorption coefficients α. According to the literature, a small interconnected porosity at the surface of porous materials leads to a very selective peak at low frequencies, which explains the abovementioned changes in the sound absorption curve characteristics [33]. Taking into account the negligible differences in the density of the foams without and with FR, this effect may be related to a modification of the polyurethane stiffness, a slightly larger number of open cells, or a more developed internal surface of the cells in the case of fire-retarded PUR. In the case of the foams with a dominating content of bio-polyol and a flame retardant, a slightly different shape of the sound absorption curve α(f) was observed than for the unmodified foams. The FR_OPU_60 and FR_OPU_100 foams showed a maximum sound absorption coefficient in the range between 1000 and 2000 Hz, and then a decrease of this value with an increasing sound frequency. The decrease in α above the first observed maximum in the sound absorption curve may be due to the passing of the micro-resonance of the cellular structure [34]. This effect is not related only to the cell size, because for the foam with the smallest cell size (OPU_100) a distinct local maximum in the α(f) curve in the frequency range of up to 2000 Hz was not observed, as opposed to the other foams. In contrast to the comparable cell geometry of the flame retardant foams, which contain an increased proportion of bio-polyol, in OPU_100 no decrease in the α value was observed in the high-frequency range.

**Figure 8:**
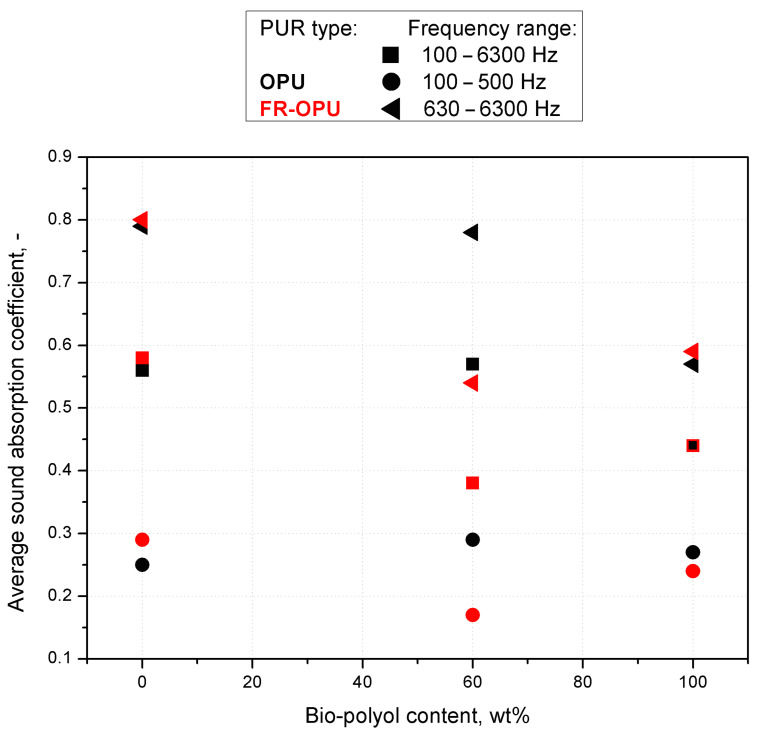
Average sound absorption coefficients of selected polyurethane foams in different frequency ranges.

In Figure 8, the values of the average sound absorption coefficient, calculated according to Equation (3), are presented. It can be noted that all the tested materials are characterized by relatively high sound absorption coefficients (0.54–0.97) in the frequency range from 1.25 Hz to 6.3 kHz. This feature is important from the point of view of potential applications of the foams as sound-absorbing materials because the abovementioned frequency range largely coincides with the band of the greatest sensitivity of human hearing (2–5 kHz). It can be stated that not only the open-cell content and size but also the addition of triethyl phosphate, which probably has an effect on the interior cell morphology, influences the air-flow resistivity and overall acoustic properties of the foam [25]. It is assumed that when the average sound absorption coefficient is more than 0.2, the material can be classified as a sound-absorbing material dedicated to building insulation [35]. All the materials tested exhibited the coefficients greater than 0.2 in the 100–6300 Hz frequency range, which allows qualifying both types of PUR foams as materials of acoustic insulation potential.

## 4. Conclusions

Open-cell polyurethane foams were manufactured and next tested in terms of their acoustic thermal insulating and mechanical properties. The foams were prepared by replacement of 20, 40, 60, 80 and 100% of a petrochemical polyol with a bio-polyol based on used cooking oil. In the second stage, a TEP flame retardant was introduced into the formulations of the modified materials. Replacing the petrochemical polyol with the bio-polyol based on used cooking oil decreased the cell diameter nearly five times. The applied flame retardant, which additionally plasticized the polyurethane matrix, had an even greater effect on reducing the diameter of the cells, especially in the case of the cross-section perpendicular to the foam rise.

It should be emphasized that for final products in the form of insulating layers used in construction, it is expected to achieve both the highest thermal insulation, and acoustic attenuation. The tests carried out in this work revealed different trends in the changes of these properties. Ideally, the most favorable properties would be displayed by a foam with the lowest possible thermal conductivity and the highest possible sound absorption coefficient. In the case of the materials under consideration, these properties change differently depending on the foam structure, in consequence of the bio-polyol addition. The most advantageous thermal properties were found for the samples containing a flame retardant with the highest share of bio-polyol (38.94 mW/m·K) and those materials were characterized by the smallest sizes of open cells. In terms of acoustic properties, the materials with large cell sizes, i.e., those without the bio-polyol, exhibited better sound absorption compared to the foams based on the bio-polyol only, especially in the frequency range of above 400 Hz. The normalized compressive strength values confirmed improved mechanical properties of the foams as an effect of applying the bio-polyol based on used cooking oil. The highest values were obtained for the materials containing the flame retardant and modified with the bio-polyols in an amount of 60%. However, all the foams based on the system with the flame retardant and modified with the bio-polyol had better mechanical properties than the reference material. Lowering the measurement temperature from 20 °C to minus 10 °C had no significant effect on the mechanical properties of the foams.

It can be concluded that obtaining the final product characterized by good acoustic and thermal properties will involve a compromise regarding the weight of both parameters depending on the final application.

## Figures and Tables

**Table 1:** Formulations of foams.

Component, g	OPU_0	OPU_20	OPU_40	OPU_60	OPU_80	OPU_100
Rokopol551	100	80	60	40	20	0
Bio-polyol	0	20	40	60	80	100
Kosmos 19	1					
Polycat 37	2					
Tegostab 8870	1.5					
Ortegol500	0.5					
Water	15					
Ongronat 2100	330.5	321.7	312.9	304.1	295.3	286.6

Formulations containing additionally flame retardant triethyl phosphate (TEP) in an amount of 30 g have letters “FR” in their names.

**Table 2:** Apparent density and coefficient of thermal conductivity of open-cell PUR foams.

Sample	d, kg/m^3^	λ 0 °C, mW/m·K	λ 10 °C, mW/m·K	λ 20 °C, mW/m·K
**OPU_0**	18.7 ± 1.34	60.32 ± 0.54	64.27 ± 0.49	69.98 ± 0.50
**OPU_20**	16.6 ± 0.27	43.00 ± 0.33	45.55 ± 0.37	48.47 ± 0.30
**OPU_40**	18.8 ± 0.10	46.04 ± 0.15	47.66 ± 0.28	51.79 ± 0.25
**OPU_60**	20.7 ± 1.70	42.15 ± 0.16	44.68 ± 0.45	47.24 ± 0.27
**OPU_80**	30.8 ± 3.18	38.35 ± 0.63	39.65 ± 0.40	42.35 ± 0.67
**OPU_100**	28.4 ± 0.84	38.08 ± 0.64	39.33 ± 0.13	42.01 ± 0.41
**FR_OPU_0**	19.6 ± 1.20	51.16 ± 0.87	54.65 ± 0.96	58.14 ± 0.01
**FR_OPU_20**	18.5 ± 0.28	39.09 ± 0.18	41.23 ± 0.04	43.35 ± 0.01
**FR_OPU_40**	18.8 ± 0.13	39.05 ± 0.69	40.95 ± 0.81	43.07 ± 0.87
**FR_OPU_60**	19.1 ± 0.36	38.21 ± 0.06	40.47 ± 0.28	42.38 ± 0.21
**FR_OPU_80**	20.8 ± 1.16	37.60 ± 0.28	38.95 ± 1.00	40.75 ± 0.94
**FR_OPU_100**	20.1 ± 1.41	37.13 ± 0.50	38.94 ± 0.71	40.89 ± 0.28

**Table 3:** Equivalent diameter and content of closed cells of PUR foams.

Sample	Equivalent Diameter, µm		Content of Closed Cells, %
	Cross-Section Area Parallel to Foaming Direction	Cross-Section Area Perpendicular to Foaming Direction	
**OPU_0**	2936 ±1 53	2673 ± 296	<5
**OPU_20**	1508 ± 202	1929 ± 416	<5
**OPU_40**	2277 ± 260	1713 ± 392	<5
**OPU_60**	1766 ± 364	1734 ± 19	<5
**OPU_80**	995 ± 162	920 ± 165	<5
**OPU_100**	630 ± 131	581 ± 143	<5
**FR_OPU_0**	2512 ± 361	2381 ± 354	<5
**FR_OPU_20**	1126 ± 221	1037 ± 205	<5
**FR_OPU_40**	1110 ± 138	813 ± 200	<5
**FR_OPU_60**	932 ± 165	803 ± 159	<5
FR_OPU_80	925 ± 213	789 ± 133	<5
**FR_OPU_100**	713 ± 140	538 ± 122	<5
